# Impact of the Umoyo mother-infant pair model on HIV-positive mothers’ social support, perceived stigma and 12-month retention of their HIV-exposed infants in PMTCT care: evidence from a cluster randomized controlled trial in Zambia

**DOI:** 10.1186/s13063-019-3617-8

**Published:** 2019-08-15

**Authors:** Sydney Chauwa Phiri, Sandra Mudhune, Margaret L. Prust, Prudence Haimbe, Hilda Shakwelele, Tina Chisenga, Mwangelwa Mubiana-Mbewe, Maureen Mzumara, Elizabeth McCarthy, Marta R. Prescott

**Affiliations:** 1 Clinton Health Access Initiative, Lusaka, Zambia; 2 Clinton Health Access Initiative, Boston, MA USA; 3 Ministry of Health Zambia, Lusaka, Zambia; 4 Center for Infectious Disease Research in Zambia, Lusaka, Zambia

**Keywords:** Mother-infant pair clinic, Social support, Retention, Stigma, HIV-exposed infant

## Abstract

**Background:**

Public health systems in resource-constrained settings have a critical role to play in the elimination of HIV transmission but are often financially constrained. This study is an evaluation of a mother-infant-pair model called “Umoyo,” which was designed to be low cost and scalable in a public health system. Facilities with the Umoyo model dedicate a clinic day to provide services to only HIV-exposed infants (HEIs) and their mothers. Such models are in operation with reported success in Zambia but have not been rigorously tested. This work establishes whether the Umoyo model would improve 12-month retention of HEIs.

**Methods:**

A cluster randomized trial including 28 facilities was conducted across two provinces of Zambia to investigate the impact on 12-month retention of HEIs in care. These facilities were offering Prevention of Mother-to-Child-Transmission (PMTCT) services and supported by the same implementing partner. Randomization was achieved by use of the covariate-constrained optimization technique. Secondary outcomes included the impact of Umoyo clinics on social support and perceived HIV stigma among mothers. For each of the outcomes, a difference-in-difference analysis was conducted at the facility level using the unweighted *t* test.

**Results:**

From 13 control (12-month retention at endline: 45%) and 11 intervention facilities (12-month retention at endline: 33%), it was found that Umoyo clinics had no impact on 12-month retention of HEIs in the *t* test (− 11%; 99% CI − 40.1%, 17.2%). Regarding social support and stigma, the un-weighted *t* test showed no impact though sensitivity tests showed that Umoyo had an impact on increasing social support (0.31; 99% CI 0.08, 0.54) and reducing perceived stigma from health care workers (− 0.27; 99% CI − 0.46, − 0.08).

**Conclusion:**

The Umoyo approach of having a dedicated clinic day for HEIs and their mothers did not improve retention of HEIs though there are indications that it can increase social support among mothers and reduce stigma. Without further support to the underlying health system, based on the evidence generated through this evaluation, the Umoyo clinic day approach on its own is not considered an effective intervention to increase retention of HIV-exposed infants.

**Trial registration:**

Pan African Clinical Trial Registry, ID: PACTR201702001970148. Prospectively registered on 13 January 2017.

**Electronic supplementary material:**

The online version of this article (10.1186/s13063-019-3617-8) contains supplementary material, which is available to authorized users.

## Introduction

The goal of eliminating vertical human immunodeficiency virus (HIV) transmission is embedded within the Joint United Nations Programme on HIV/AIDS (UNAIDS) 90–90-90 targets which are to be achieved by 2020 [1]. Taking antiretroviral therapy (ART) during pregnancy and breastfeeding can significantly suppress the HIV viral load of a pregnant woman, reducing the risks of mother-to-child transmission of HIV to negligible levels [2]. However, more than half of mother-to-child transmissions occur during breastfeeding, signalling the need for more attention on postpartum follow-up and retention in care during breastfeeding [3].

HIV stigma and discrimination are two important detractors to the elimination of mother-to-child transmission of HIV as they are strong barriers against accessing HIV prevention, testing, and treatment services [4–7]. Abundant evidence shows that people living with HIV/AIDS are better able to cope with the negative health stressors of discrimination and stigma in the presence of social support as it has been associated with positive health outcomes for such as self-efficacy, adherence to ART, quality of life, physical health, and mental health [8–13]. Indeed, the WHO has recognized that people living with HIV/AIDS have important psychosocial needs that need to be integrated into HIV care such as prevention and treatment of mental health disorders, assistance on coping with discrimination and stigma and financial forms of assistance [14].

While the vast majority of research linking ART adherence to social support has been conducted on adults, little is known about the relationship between social support for mothers and health outcomes of their HIV-exposed infants (HEIs). Interventions focused on structured peer-support models such as the Mother2mother mentor program have been implemented in selected African countries though evidence on the impact of social support on retention of HEIs along the Prevention of Mother-to-Child Transmission (PMTCT) cascade is promising but mixed [15–17].

In resource-constrained settings like Zambia, significant strides have been made in increasing PMTCT service utilization and coverage with the national roll out of WHO Option B+ in 2013. However, more needs to be done to achieve elimination of mother-to-child transmission. Nationwide data on the retention rates of mother-infant pair (MIP) cohorts within PMTCT are not available but overall, HIV-positivity rates for early infant diagnostic tests are increasing in infant age from those that test at 6 weeks up to 24 months [19]. Efforts to eliminate mother-to-child transmission of HIV are constrained by a plethora of challenges including but not limited to limited human resources for health; prolonged turnaround times for early infant diagnosis test results; poor 24-month retention, weak cohort-monitoring systems for tracking MIPs; and limited community support systems [20]. Additionally, the standard of PMTCT care in Zambia is a potential barrier to improved retention because mothers of HEIs attend monthly under-five clinics together with HIV-negative mothers. Anecdotally, mothers of HEIs receiving additional services (e.g., prophylaxis and counseling) beyond what HIV-negative mothers are receiving can betray the HIV statuses of the different mothers. Further, to avoid speculation on HIV statuses by offering different services to mothers depending on their status, health workers sometimes request mothers of HEIs to wait until all HIV-negative mothers have been attended to, in order to receive their HIV-specific services. However, the fact that these positive mothers stay longer at the clinic for a routine under-five clinic is also a fuel for additional speculation from their HIV-negative peer mothers who are released early.

Within the context of constrained budgets and competing health needs, promising interventions, such as the Mother2mother program, are unlikely to be prioritized for support by the Zambian Ministry of Health (MOH) due to the financial costs. Alternative approaches that can be feasibly implemented by public health systems are needed. In Zambia, there is an MIP clinic model being implemented in selected public health facilities in two districts (Kitwe in Copperbelt Province and Chipata in Eastern Province). This MIP clinic approach, called Umoyo which is translated as “Clinic of Life” from a local Zambian dialect, is being sustained by a lean staff of health workers supported by few volunteers without external financing or material support outside what is received from the government. In Umoyo clinics, under-five clinics are scheduled in such a way that HIV-positive mothers and their HEIs receive integrated HIV and routine post-natal care services on a day separate from HIV-negative mothers. Therefore, a Umoyo MIP clinic ensures that MIPs receive the services that they need and fosters the development of informal mother-to-mother peer-support systems.

The Zambian MOH was interested in evidence on whether Umoyo clinics could improve retention of HEIs in care for possible scale-up. However, only anecdotal evidence existed in support of this model and, therefore, a rigorous evaluation of this clinic model was necessary to inform policy-makers. Therefore, this study was commissioned to answer the question: “What is the impact of Umoyo MIP clinics on retention of HIV-exposed infants in PMTCT care at 12 months after birth?”

## Methods

### Study design

This impact evaluation was a two-arm, difference-in-difference, cluster randomized controlled trial conducted in Lusaka and Eastern provinces of Zambia. It assessed the change in proportion of HEIs retained in care 12 months before and after the Umoyo MIP clinic was launched in 14 intervention sites compared to the change in retention in 14 control sites with the standard of care.

### Study population

There were three main target populations from which the sample was drawn: health facilities, HEIs and HIV-positive mothers.

Facilities were eligible for the study if they were public sector health facilities caring for large numbers of HIV-positive pregnant women (i.e., an estimate of 10 HEIs born per month) and supported by the implementing partner for the study (the Center for Infectious Disease Research in Zambia (CIDRZ)) at the launch of Umoyo in February 2017. At that time, CIDRZ was supporting a total of 124 public health facilities in Lusaka, Western and Eastern provinces with the goal of expanding access to more efficacious PMTCT regimens and antiretroviral therapy (ART), and on early infant diagnosis for HEIs.

The HEI target populations for the study (the primary cohort) were those infants born to HIV-positive mothers within a 4-month period (November 2015 through February 2016 for the pre-implementation period or November 2016 through February 2017 for the post-implementation period) and had their 6-week early infant diagnostic test as well as their first follow-up visit at the public health facilities.

The final target population was HIV-positive mothers to participate in an exit survey, and mothers were eligible if they had attended the public health facilities for infant care (6 weeks to 2 years old) during the study period.

### Sample size

A cluster randomized controlled trial was conducted in a sample of 28 facilities. Each of the facilities were selected based on availability of PMTCT services and approximately 10 or more HEIs born each month. The sample of HEIs was selected from the HEI registers based on the inclusion criteria in Table 1. All the criteria in the table need to be jointly met for a child to be considered eligible for enrolment in the study.

**Table 1:** Sample selection for children

Selected characteristics	Pre implementation	Post implementation
Birth	1 Nov 2015–31 Mar 2016	1 Nov 2016–31 Mar 2017
Age at 6-week DNA PCR test	4 to 12 weeks old	4 to 12 weeks old
Date of 6-week DNA PCR test	1 Jan 2016–30 Apr 2016	1 Jan 2017–30 Apr 2017
Follow-up 10-week visit	Yes	Yes

Note: dates are all inclusive

*DNA PCR* deoxyribonucleic acid polyerase chain reaction

For the primary cohort, we report the a priori estimated effect size we could detect assuming 14 facilities in each of two arms and 30 MIPs per facility (pre and post). The following equation [21] with the specific parameters in Table 2 was used to estimate that we would be able to determine a

$$ c\kern0.5em =\kern0.5em 1\kern0.5em +\kern0.5em {\left({z}_{\upalpha /2\kern0.5em }+\kern0.5em {z}_{\upbeta}\right)}^2\frac{\left[{\uppi}_0\left(1-{\uppi}_0\right)+\kern0.5em {\uppi}_1\left(1-{\uppi}_1\right)\right]\times \left[1+\left(m-1\right)\uprho \right]}{m{\left({\uppi}_0-{\uppi}_1\right)}^2} $$

**Table 2:** Sample size calculation for primary outcome

*α* (alpha)	0.05
*β* (power)	0.80
*π*_*o*_ (average change in control facilities)	0.01
*π*_1_ (average change in intervention facilities)	0.13
*m* (mean number of eligible MIPs per facility)	30
*ρ* (rho)^a^	0.15
*C* (total facilities included per arm)	14

^a^a conservative value of rho was used

*MIP* mother-infant pair

12% difference in the average change in the proportion of HEIs who returned for their 12-month visit (alpha 0.05 and power of 80%).

For the mother interviews, mothers were approached during clinic days when PMTCT services were offered: women were eligible if they were the mother of an HEI born within 24 months of the survey date, and if the mothers were over the age of 18 years. Women and children who did not meet these criteria or those that did not attend the under-five clinic on the day the research team visited the facility were excluded.

### Intervention

CIDRZ was providing intensified on-site mentorship to facility staff on provision of integrated services to HIV patients in general. Additionally, health facility staff and community health workers were oriented and mentored on early infant diagnosis, family planning, and how to strengthen documentation in the health facility registers. Although CIDRZ was mentoring facility staff on provision of integrated services to HIV patients, there was no specific intervention targeting MIPs in PMTCT care. However, the community health workers were actively following up MIPs who missed scheduled appointments.

The Umoyo MIP clinic is a designated clinic day for HIV-positive mothers and their HEIs where they receive routine child health care and routine maternal ART services including the following:

- Enhanced, group-based sensitization and intensified Information Education and Counseling for mothers
- Integrated services including HIV and tuberculosis (TB) screening for mothers and infants, provision of isoniazid TB prophylaxis for eligible mothers and infants; family planning for mothers, and immunizations and ART services for infants and
- Active defaulter tracing by lay counselors

Services are provided to eligible MIPs on one or more days in the month, depending on the volume of infants that the health facility expects in the month. Facilities with relatively large catchment populations had more than one Umoyo MIP clinic day in a month – even up to four per month in one site. Regardless of the number of sessions that a clinic held, each mother attended only one visit monthly.

Whereas the primary population for the study was a narrow cohort of infants in 2016 and 2017, the population eligible for the intervention was any infant born to an HIV-positive mother all the way up to 24 months of age. In addition, MIPs can remain in the Umoyo MIP clinic until cessation of breastfeeding and/or the child is 24 months. Those infants who test HIV-negative at 24 months are discharged from PMTCT care while those who seroconvert are transitioned to continue treatment at the normal ART clinic together with their mothers. However, only results on the primary cohort of infants are presented in this study.

Six weeks before the study began, a 2-day off-site orientation of at least two health facility staff in the treatment arm was conducted by staff from Kitwe District who were already implementing Umoyo within the public health system. A follow-up on-site (facility by facility) orientation of all health facility including lay counselors was conducted by the CIDRZ team. To ensure that data quality was maintained in both study arms, the CIDRZ team conducted data-quality mentorship exercises in all facilities enrolled in the study. Additionally, across both study arms, a nurse within mother and child health department was identified to be the custodian of the list of defaulters and was responsible for communicating with the lay counselors regarding any MIPs that needed follow-up within the community. This on-site orientation marked the beginning of the first month of the Umoyo MIP clinic implementation. Throughout the intervention, the CIDRZ role was to monitor the implementation fidelity of intervention (at least once within a quarter), ensure that each facility has adequate supplies of requisite medical supplies needed for PMTCT service provision and mentorship on data quality.

Intervention sites were also provided with a job aid (Additional file 1) to help the health facility staff with scheduling their monthly Umoyo MIP clinics. Intervention sites were also instructed to keep an attendance book (Additional file 2) for the Umoyo program. At the control sites, the facilities continued to function on a business as usual basis.

### Study outcomes

The primary outcome of interest was the change in the proportion of HEIs who were retained in care at 12 months; this outcome combined HEIs with negative or unknown HIV status receiving a test at 12 months with HIV-positive infants attending the scheduled 12-month visit for ARV drug refills. Secondary outcomes included:

- Change in the proportion of HEIs retained at 6 months
- Change in the proportion of HEIs with regular attendance as defined by retention in care at 6, 9, and 12 months of age
- Change in perceived social support of mothers with HEIs and
- Change in perceived stigma of mothers with HEIs

### Randomization

Given the relatively small number of facilities per arm (*N* = 14), a covariate-constrained optimization technique was used to randomize intervention assignment and achieve balance in the two arms [22]. Using estimates for annual expected HEI live births, the 2016 catchment population and districts, facilities were randomized using covariate-constrained randomization. The 28 sites were randomized 1000 times and those iterations in which there was imbalance with an alpha of 0.1 on the aforementioned characteristics were removed. Among those remaining iterations where there was no imbalance detected at a threshold (F test beta = 0, alpha 0.1), an iteration of assignment was then selected at random.

### Data collection

Three key sources were collected: patient registers, mother exit interviews, and facility-level questionnaires. We also gathered data on monthly visits using the *Septrine/Cotrimoxazole booklet*s (often called *Septrine booklets* in Zambia) and information on TB treatment using the *Zambia National TB & Leprosy Control Program, IPT Register*. However, we found that these booklets were not utilized in a standard way across all facilities. Thus, to minimize bias, the outcomes within this report rely on the *HIV Exposed Infant (and Mothers) Follow-Up Register* only. We list our specific method of defining “retained” for each outcome by HIV status within the “Analysis” section below.

For the mothers’ stigma and social support, we performed mother exit surveys using questionnaires validated in African countries with comparable HIV profiles to Zambia such as Swaziland, Lesotho Malawi, Tanzania, and Uganda [23, 24]. The tools were translated into local dialect and back-translated by an independent contractor. The surveys were created as follows:

- *Social support*: Nine questions were adapted from the Social Provisions Scale [23] to assess the degree of social support that women received from other women that attend the standard of care under-five clinic (pre implementation) or the Umoyo MIP clinic (post implementation). Adaptations reduced the scale to three items (“Disagree,” “Not sure,” and “Agree”) and included an option for “Don’t know, refused or don’t want to say”
- *Stigma*: 28 questions on stigma have been adapted from the HIV/AIDS stigma instrument (HASI-P) to include questions on internalized stigma and enacted stigma (e.g., verbal, fear of contagion, social isolation, and workplace stigma) [24]. We adapted the scale to limit to three (rather than four options), “Never,” “Once or twice,” and “Several times”; we also included an option for “Don’t know/refused/not applicable.” Questions regarding health care worker (HCW) stigma were added and had a similar coding

Facility level data was collected from surveys with key facility-level personnel to assess the general working environment in the facilities during the period of the study. The aspects relating to the general working environment included frequency of stock-outs for key commodities relevant to the study outcomes, and whether sites received any additional support for PMTCT and/or pediatric HIV from implementing partners other than CIDRZ.

Data was collected at three points in time, with training of data collectors immediately prior to the start of each data collection (Table 3).

**Table 3:** Schedule of data collection

Period	Purpose	Training dates	Number of data collectors	Entire data collection
First	Pre-implementation data; mothers’ survey; facility information	14 Feb 2017–17 Feb 2017	12	20 Feb 2017–10 Mar2017
Second	Post-implementation data	30 Aug 2017–1 Sept 2017	8	4 Sept 2017–20 Sept 2017
Third	Post-implementation data; mothers’ survey; facility information	4 Apr 2017–6 Apr 2017	14	9 Apr 2018–11 May 2018

Data collectors were hired specifically for this study and received 3-day training. Direct supervision within facilities was conducted for all enumeration teams on a rolling basis and troubleshooting support was provided to all enumerators. All data was electronically entered using SurveyCTO at the point of collection except the mother interviews which were administered using paper tools. At the end of the data collection period, all the mothers’ questionnaires were entered twice by two different enumerators. Once the double data entry was complete, the discrepancies in entry were reconciled by checking with the entry in the hardcopy.

### Analysis

Facility, child, and mother characteristics were compared to examine balance between the two arms before the intervention was implemented. For the facility characteristics, the self-reported values from the HCW were assumed to be facility-specific and the distribution (percentage and 99% confidence interval (CI)) was calculated. For the child and mother characteristics, an average estimate was calculated for each facility which was then pooled together per arm; to this end, the values, unless otherwise specified, are aggregate facility-level values. This analysis method for cRCTs produces a conservative estimate of difference when the facility sample size is less than 10 facilities per arm [6]. The difference between the two arms during the pre-implementation phase, as well as in the change from pre to post implementation was tested using unweighted *t* tests with unequal variances.

Given that the evaluation was being implemented in a natural setting, monitoring of Umoyo exposure was not standardized or rigorously maintained; thus, per protocol analysis was not carried out. All analysis reported are intent-to-treat.

For the outcomes, we categorized the eligible children from both the pre-implementation period and post-implementation period as retained according to the outcomes as listed in Table 4.

**Table 4:** Categorization for primary child outcomes

Outcomes	HIV-exposed and -negative	HIV-positive
Change in the proportion of HEIs who were retained in care at 12 months of age	Evidence of 12-month test • Date of 12-month test date listed; or • Result of 12-month rapid diagnostic test result listed (i.e., positive or negative)	• Attended 12-month visit if tested positive at 6-week; or • Attended 12-month visit (i.e., 6-month ARV registry) if tested positive at 6 months
Change in the proportion of HEIs with regular attendance and retained in care at 6, 9, and 12 months of age	Evidence 6-month test occurred: • Date of either 6-month virologic test date listed; virologic results received listed; or virologic test results collected by mother; or • Result of 6-month virologic test result listed *and* evidence attended 9-month session • Recorded whether the infant was either breastfeeding, received cotrimoxazole, or received nevirapine at 9-month visit *and* evidence of 12-month test • Date of 12-month test date listed; or • Result of 12-month rapid diagnostic test result listed (i.e., positive or negative)	• Attended 6-, 9-, and 12-month visit, if tested positive at 6 weeks; or • Attended 12- and 9-month visit (i.e., 6- and 3-month ARV registry) if tested positive at 6 months

*ARV* antiretroviral therapy, *HEI* HIV-exposed infant

For each outcome above, an individual-level logistic generalized estimating equation (GEE) with a binomial distribution and link logit was conducted as part of the sensitivity analysis which accounted for clustering and varying number of eligible children per facility. This regression included an indicator variable for time (pre vs. post), an indicator for whether the child came from an intervention facility (control vs. intervention), and a time by intervention interaction to estimate the program impact. The model also accounted for a potential difference during the pre-implementation phase between the arms; though differences were not statistically significant, we included a covariate for the average number of children per facility.

To account for the multiple outcomes and increased risk of Type I error (incorrectly rejecting the null hypothesis), we adjusted the alpha to an alpha of 0.05/11 (for 11 outcomes) or 0.0045; thus, we report 99% CIs (1–0.005) for our estimates, and all *p* value testing is considered significant if below < 0.0045. The GEE model included the facility-level as cluster with an exchangeable correlation structure.

For mothers regarding social support and stigma outcomes all mothers received a score similar to the Holzmer 2007 work but accounting for the fact we included a “not applicable” (“N/A”) response and had a lower maximum possible range [24]. To this end, we gave all “Don’t know, refused, or don’t want to say, or N/A” responses a missing value. We then gave those who had the lowest answer on the item (e.g., disagree, or never occurred, or no) a zero and those with the next two levels of responses a 1 or 2, accordingly. We then created a weighted score: all items for the specific topic were summed and the mother received a score by dividing this sum by the total number of non-missing items. Thus, the score always represented the highest level of either social support or stigma. Similar to the child outcomes, the analysis was run in two methods: a facility-aggregated analysis using unweighted *t* tests and an individual-level linear GEE. For the multivariable linear GEE model, though differences were not statistically significant, we included covariates for the average number of interviews per facility, if the mother was aged over 40 years, if she were married, or had been on HIV treatment for a year.

## Results

**Fig. 1:**
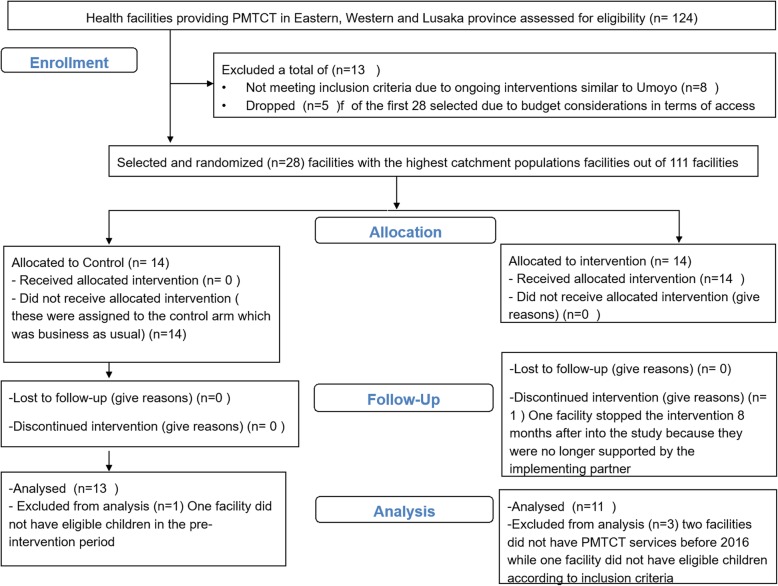
Final sample of facilities

Figure 1 below illustrates the final sample of facilities obtained for the study.

Of the original 124 public facilities that were supported by our implementing partner (CIDRZ), eight were removed because of ongoing interventions similar to the Umoyo program. Among the remaining 116 facilities, facilities were ranked on their catchment population, and the 28 facilities were selected (across the three provinces and eight districts) that had the largest catchment populations and thus likely the largest HIV client population. Within the original 28 selected, five were replaced due to logistical considerations. Specifically, to reduce the budget and logistical challenges, districts where at least one other facility had been selected were included in the sample; these original five facilities were from districts where only one facility was selected. The final sample was 28 facilities across two provinces (Eastern and Lusaka) and seven districts (Fig. 1).

Table 5 displays the breakdown of our study sample for the facilities, children, and mothers.

**Table 5:** Distribution of selected characteristics of the study sample at the facility, primary cohort, and mother level

	Pre implementation		Post implementation		*P* value
	Control	Intervention	Control	Intervention	
Facility-level characteristics					
Total facilities in study	14	14	14	14	
Total facilities with facility survey	12	10	14	11	
Proportion with frequent stockouts of DBS in the past 12 months (%, 95% CI)	0	0	7.1 (−8.3, 22.6)	9.1 (−11.2, 29.3)	0.9
Proportion with an NGO is working in the catchment area (%, 95% CI)	91.7 (73.3, 110.0)	100 (100,100)	100 (100,100)	100 (100,100)	0.4
Child cohort characteristics					
Total facilities	14	11	14	11	
Total children	166	415	224	368	
Average number of children per facility (mean, 95% CI)	11.9 (2.6, 21.1) Min: 1 Max: 52	37.7 (−1.6, 77.1) Min: 2 Max: 195	16.0 (5.9, 26.1) Min: 1 Max: 56	33.5 (−3.3, 70.2) Min: 2 Max: 192	0.1
Proportion of children born within specific month (facility-aggregated average, 95% CI)					
November birth	12.3 (1.8, 22.9)	15.0 (5.3, 24.6)	25.6 (10.8, 40.5)	14.3 (4.3, 24.3)	0.05
December birth	36.3 (23.8, 48.7)	32.5 (19.6, 45.4)	22.9 (13.2, 32.5)	29.8 (12.0, 47.5)	0.4
January birth	27.9 (12.6, 43.2)	22.9 (10.8, 35.0)	24.6 (14.2, 34.9)	27.3 (14.0, 40.7)	0.5
February birth	23.5 (6.7, 40.2)	29.6 (14.3, 45.0)	26.9 (10.4, 43.4)	28.5 (10.7, 46.3)	0.8
Mean age in days at 6–10-week HIV test	52.0 (47.2, 56.7)	49.7 (46.2, 53.9)	56.4 (51.1, 61.7)	49.0 (45.0, 52.9)	0.2
Proportion of girls within facility	48.9 (29.6, 68.2)	49.7 (41.7, 57.6)	44.5 (36.3, 52.7)	61.5 (50.8, 72.1)	0.2
Mother characteristics					
Total facility	11	11	14	11	
Average number of interviews per facility	6.9 (3.9, 9.9)	10.7 (7.5, 13.9)	13.7 (10.7, 16.7)	11.9 (8.5, 15.4)	0.02
Over the age of 40 years	6.0 (0.16, 11.9)	12.8 (3.8, 21.8)	7.5 (1.4, 13.6)	13.1 (5.3, 21.0)	0.7
Currently married	84.0 (74.9, 93.1)	88.1 (81.4, 94.7)	77.6 (69.1, 86.1)	83.2 (75.2, 91.2)	0.6
On HIV therapy for 1 year	68.9 (49.6, 88.2)	78.4 (69.1, 87.8)	71.7 (64.3, 79.0)	78.7 (65.5, 91.8)	1.0

*CI* confidence interval, *HIV* human immunodeficiency virus, *NGO* non-governmental organization

Of the 28 facilities, not all facilities had facility-characteristic data collected at both time points. Two of the pre-implementation facility surveys, while collected, were not entered into the system at the time. Overall, no facilities reported a stock out in the year before Umoyo, but there were reports of stock outs of dried-blood-spot test kits after Umoyo implementation with 10% of control facilities and 20% of intervention facilities reporting a stock out in the 12 months before the endline survey. The affected facilities which experienced stock outs are found in a similar geographical region which was had overall challenges with stock supply for a short period.

For the child-level analysis, we report the statistics only among the facilities used in the final analysis (14 control and 11 intervention): two facilities did not have ART services in 2016 (pre implementation), and one facility did not have any eligible children for the study in 2017 (post intervention). During the pre-implementation phase, the average number of children in the control arm was 11.9 children per facility (95% CI 2.6, 21.1) while the average for the intervention arm was 37.7 per facility (95% CI − 1.6, 77.1). There was large variation in the number of children per facility as evidenced by the relatively large CIs for facilities in both the control and intervention arms. There was no evidence of a difference in the change of eligible children per facility between the two arms at an alpha of 0.01 (*p* value: 0.1). During the pre-implementation phase, eligible infants in the control arm were roughly 52 days old (95% CI 47.2, 56.7) when they received their 6-week HIV test while eligible children in the intervention arm were 50 days old (95% CI 46.2, 53.9); there was no evidence of a difference in the change of mean age at testing between the two arms (*p* value 0.2).

In Table 6, we report the child-level outcomes for the outcomes of interest.

**Table 6:** Distribution (% and 99% confidence interval (CI)) and comparison of child-level outcomes

	Pre implementation		Post implementation		Difference in difference over time (99% CI)	*p* value from *t* test
	Control	Intervention	Control	Intervention		
Total facilities	14	11	14	11		
Total children enrolled in study	166	415	224	368		
Proportion of eligible children retained in care at 12-month visit	46.2% (19.8%, 72.5%)	45.7% (13.4%, 77.9%)	45.0% (18.1%, 71.9%)	33.1% (3.8%, 62.2%)	− 11% (− 40.1%, 17.2%)	0.3^a^
Proportion of eligible children retained in care at 6-, 9-, and 12-month visits	28.8% (5.0%, 52.5%)	33.2% (3.6%, 62.8%)	28.2% (11.6%, 44.7%)	26.1% (−0.02%, 54.3%)	− 6.5% (− 24.7%, 11.7%)	0.3

^a^Removed the facilities that are not eligible in the pre-intervention (Kazimva and Kanyama West) or post-intervention (Madzimawe) periods

We found that the Umoyo program did not improve the proportion of children who were retained in care at 12 months (− 11%; 99% CI −40.1%, 17.2%) or continually from 6 to 12 months (− 6.5; 99% CI − 24.7%, 11.7%). In logistic GEE models, evidence even suggested that the Umoyo program may have had a significantly negative impact on retention as the treatment effect over time (i.e., interaction term) showed a reduction of 0.2 lower odds of retention at 12 months from baseline to endline (99% CI 0.1, 0.4) as well as a 0.3 lower odds of retention at 12 months (with 6- and 9-month retention) from baseline to endline for children enrolled in an Umoyo facility as compared to the change for children in control facilities (Table 7).

**Table 7:** Generalized estimating equation (GEE) model results for primary cohort

	12-month retention	6-month retention	12-month retention (with 6 months and 9 months)
	aOR and 99% CI	aOR and 99% CI	aOR and 99% CI
Total facilities	25	25	25
Total children	1173	1173	1173
Time			
Pre intervention	Ref	Ref	Ref
Post intervention	1.3 (0.8, 2.3)	1.5 (0.8, 2.4)	1.1 (0.6, 2.0)
Treatment			
Control	Ref	Ref	Ref
Intervention	2.1 (1.1, 4.1)	2.1 (1.2, 3.8)	2.2 (1.1, 4.3)
Interaction time*treatment	0.2 (0.1, 0.4)	0.8 (0.4, 1.6)	0.3 (0.2, 0.7)
Number of children per facility	1.0 (0.99, 1.00)	1.0 (0.99, 1.00)	1.0 (0.99, 1.00)

*aOR* adjusted odds ratio, *CI* confidence interval

Table 8 shows distribution of the mothers’ outcomes on stigma and social support for mother exit interviews. Over time, according to an alpha of < 0.01, we did not see evidence that the Umoyo program improved the Social Support Scores (mean 0.2, 99% CI − 0.4, 0.8), enacted stigma (mean − 0.1, 99% CI − 0.3, 0.01), HCW stigma (mean − 0.2, 99% CI − 0.5, 0.03), or the internalized stigma (mean 0.01, 99% CI − 0.3, 0.3).

**Table 8:** Distribution (mean and 99% confidence interval (CI)) and comparison of outcomes for mothers’ Social Support and Stigma Scores

	Pre implementation		Post implementation		Difference in difference	*P* value from *t* test
	Control	Intervention	Control	Intervention		
Total facilities^a^	11	11	11	11		
Social Support Scale (0–2)	1.60 (1.39, 1.82)	1.30 (1.01, 1.58)	1.46 (1.22, 1.69)	1.37 (0.91, 1.84)	0.22 (− 0.36, 0.81)	0.3
Stigma enacted (0–2)	0.08 (0.001, 0.15)	0.12 (0.06, 0.18)	0.17 (0.07, 0.27)	0.07 (− 0.004, 0.15)	− 0.14 (− 0.29, 0.01)	0.02
Stigma HCW (0–2)	0.49 (0.30, 0.69)	0.64 (0.47, 0.81)	0.58 (0.44, 0.73)	0.49 (0.34, 0.64)	− 0.24 (− 0.51, 0.03)	0.02
Internalized stigma (0–2)	0.53 (0.30, 0.77)	0.44 (0.29, 0.59)	0.34 (0.19, 0.48)	0.26 (0.09, 0.42)	0.01 (− 0.29, 0.32)	0.9

^a^Removed the facilities that do not have data in the pre- or post-intervention period

*HCW* health care worker

With the linear GEE models, however, we did find that the treatment effect over time; (i.e., the interaction term) showed that mothers in the intervention arm from baseline to endline increased their Social Support Score 0.31 units more as compared to the change in Social Support Score for mothers in control facilities (99% CI 0.08, 0.54). We also found that the HCW stigma decreased over time to a larger degree from baseline to endline for mothers in the intervention facilities as compared to the change in reported HCW stigma for mothers in control facilities (mean difference -0.27; 99% CI -0.46, -0.08) (Table 9).

**Table 9:** Generalized estimating equation (GEE) linear model results

	Social Support Score	Enacted Stigma Score	HCW Stigma Score	Internalized stigma
	Adjusted β	Adjusted β	Adjusted β	Adjusted β
Total facilities	22	22	22	22
Total mothers	479	479	479	479
Time				
Pre intervention	Ref	Ref	Ref	
Post intervention	− 0.09(− 0.26, 0.08)	0.05(− 0.05, 0.14)	0.14(− 0.004, 0.27)	− 0.13(− 0.31, 0.05)
Treatment				
Control	Ref	Ref	Ref	
Intervention	− 0.22(− 0.5, 0.08)	0.004(− 0.09, 0.10)	0.11(− 0.07, 0.29)	− 0.03(− 0.24, 0.17)
Interaction time*therapy	0.31 (0.08, 0.54)	− 0.09(− 0.22, 0.04)	− 0.27(− 0.46,-0.08)	0.01(− 0.23, 0.25)
Number of interviews per facility	− 0.002(− 0.03, 0.02)	0.002(− 0.004, 0.008)	0.01(− 0.003, 0.03)	0.003(− 0.01, 0.02)
Aged over 40 years	− 0.12(− 0.30, 0.07)	− 0.02(− 0.12, 0.08)	− 0.05(− 0.20, 0.09)	− 0.02(− 0.20, 0.17)
Married	− 0.09(− 0.23, 0.05)	− 0.02(− 0.10, 0.06)	0.002(− 0.11, 0.12)	0.04(− 0.11, 0.18)
On HIV treatment at least 1 year	− 0.07(− 0.21, 0.06)	0.05(− 0.03, 0.12)	− 0.02(− 0.12, 0.09)	− 0.04(− 0.18, 0.09)

## Discussion

The findings of this study show that the Umoyo MIP clinic did not have a significant impact on the 12-month postpartum retention of HEIs in PMTCT care. Rather, GEE results suggest that the 12-month retention outcomes over time for HEIs in control facilities were better than those observed in the intervention sites. At the same time, the Umoyo MIP clinic may have had a statistically significant impact on improving social support over time and reducing HCW stigma but did not have any effect on reducing internalized stigma nor enacted stigma.

The finding that the Umoyo MIP clinics did not have a significant effect on retention of HEIs has been found in cluster randomized controlled trials testing the impact of Umoyo MIP clinic-like interventions in similar settings such as Malawi and Zimbabwe. In Malawi, an MIP clinic offering integrated services to HIV-positive mothers and their HEIs on the same day including sending SMS reminders to mothers on follow-up appointments found no statistically significant impact on retention of infants in PMTCT care. The lack of impact was largely attributed to partial implementation fidelity where about 42% of the mothers were exposed to the intervention [25]. In Zimbabwe, a cluster randomized controlled trial found that mother support groups at health facilities had no statistically significant impact on retention of HEIs at 12 months [26]. However, compared to our work these studies found a higher proportion of retention in the intervention than in the control, though insignificant, while our study suggested a lower rate of retention due to the program.

Among others, such as lower power due to facility exclusion and larger than anticipated variation in facility sizes and/or that the intervention may not have had an impact, two possible additional reasons for the potentially null or negative impact observed in this study are described next. First, the evidence on exposure to the intervention was limited but shown to be relatively low at roughly 50%. Based on conversations with the facility staff and field visits, it was obvious that the intervention was implemented in non-standard ways, pointing to weak monitoring of the intervention. Even in places where strong Umoyo MIP clinic champions were present at facility level at the outset, staff turn-over led to variable content and quality of Umoyo MIP clinics. The second possible reason for the negative impact of Umoyo MIP clinics on retention of HEIs is that facility staff reported that the intervention may have had an adverse effect on data completeness of facility registers. Anecdotally, facility staff in intervention sites mentioned that they were the busiest on Umoyo clinic days due to the huge volume of services to be provided, a fact that was compounded by constrained human resources. Therefore, it is possible that staff may have de-prioritized the need to update the facility registers even though PMTCT-related services had been provided. In turn, this missing data in facility registers, may have limited our ability to examine the impact of the Umoyo model. Future versions of the model should work to include more comprehensive data management and monitoring systems. Additionally, the study team did collect additional data from secondary sources such as the TB Register, Isoniazid Register and the Cotrimoxazole Dispensing Register, these secondary registers were not uniformly used across facilities and are not reported anywhere in this manuscript as they would serve to increase bias.

The indicative positive results of the intervention on social support and HCW stigma are similar to other peer-to-peer mentor support studies because the Umoyo MIP clinics are founded on the idea that mothers interact with the mothers much more than would be the case without them [17, 18]. Additionally, since the running of Umoyo MIP clinics is HCW-driven, it is possible that a reduction in HCW stigma was observed. However, there is no literature on studies that have evaluated the evolution of stigma using the HASI-P instrument in order to compare with our study findings for non-improvement in internalized and enacted stigma.

Several lessons have been learned in the course implementing this research that other researchers may benefit from. Firstly, if resources permit, it would be valuable to validate the final sample generated from a sampling frame especially if health facility characteristics are an important part of the selection criteria and randomization. Secondly, having formal measures of implementation fidelity would have given more insights into whether the intervention was really not helpful or that the intervention was poorly implemented. Thirdly, the reliance on data from the public health system remains a challenge and while we had deliberate reasons for doing so, researchers should continue to find innovative way of collecting information that is already contained in formal registers to avoid duplication.

## Limitations

Our study has several limitations. One limitation is that three facilities in the intervention arm and one in the control arm were excluded from the difference-in-difference analysis due to lack of implementation. Thus, power was lower than anticipated and the variation in facility sizes was large; thus, our minimal effect size was lower than estimated. Moreover, the original sampling frame obtained for randomization and sample selection was not as accurate as we had anticpated; thus, we were unable to determine the risk to power ahead of time. Another limitation is that though there was no statistical difference between the two arms and examined covariates (save for the patient load per facility), there may have been residual imbalance at the time the intervention began and we were not able to collect individual-level data about other potential confounders.

This study aimed to build on existing health-system protocols and documentation without creating additional requirements or resources; however, it should be noted that completeness of the data registers was an issue. We sometimes found registers other than the primary register as alternative sources of the required information. As these secondary registers were not present in all facilities, the extent to which they had complete data compared to the primary register is unknown. Therefore, in facilities where other registers were preferred by facility staff as the primary register for recording information, then the outcomes may have been underestimated. The study also did not account for the variability in the use of the primary register across all the facilities nor did the study compare the data completeness in these secondary registers to the primary register. We do not believe, however, that this would have differed between the study arms. More worrying, however, is the anecdotal point above: that those intervention facilities may have de-prioritized accuracy of the records during busy Umoyo clinic days. Future models would benefit by creating innovative solutions to record keeping given the limited staff.

The other limitation of the study relates to the overall fidelity of the intervention. Some facilities, usually the small facilities, had near perfect fidelity to the intervention while the large-volume sites had poor fidelity. Furthermore, the study had no formal measurements of implementation fidelity. Finally, regarding the mothers’ stigma and peer-support outcomes, the limitation with these results is that they were not performed on the same mothers before and after the intervention. Therefore, their applicability is subject to the limitations of the methodology employed, i.e., confounding factors may be able to explain the presence or absence of the impact seen.

## Conclusion

The results of this cluster randomized controlled trial found that a low-cost intervention, such as the Umoyo program, had no impact on increasing retention of HEIs within PMTCT and did not reduce internalized and enacted stigma using both the GEE estimates and the unweighted t-test comparison estimates. Regarding peer-support and HIV stigma, the two estimation procedures provided different conclusions. The GEE gave support that Umoyo increases social support and reduced HCW stigma but the un-weighted *t* test showed no impact. Factors, such as low exposure to the intervention due to poor implementation fidelity, de-prioritization of filling in the primary source of data collection for this study in the intervention sites due to the burden of the intervention on Umoyo MIP clinic days, and non-use of a potential key register for the 12-month testing information, could explain the absence of impact and an association with worse outcomes for HEIs in the intervention. Alternative models that can improve retention of HEIs in PMTCT care within a public health system in resource-constrained settings need to be evaluated. If Umoyo is to be scaled, specific systems’ improvements should be made including increasing the number of HCW per facility.

## Additional files

Additional file 1: Umoyo scheduling aid. (JPG 355 kb)

Additional file 2: Sample of Umoyo attendance book. (PNG 4 kb)

## Data Availability

The datasets used during the current study are available from the corresponding author on reasonable request.

## Abbreviations

ART: Antiretroviral therapy
cRT: Cluster randomized trial
GEE: Generalized estimating equation
HCW: Health care worker
HEI: HIV-exposed infant
MIP: Mother-infant pair
PMTCT: Prevention of Mother-to-Child Transmission
WHO: World Health Organization
