# Supplementary figures and images for: Impact of the Umoyo mother-infant pair model on HIV-positive mothers’ social support, perceived stigma and 12-month retention of their HIV-exposed infants in PMTCT care: evidence from a cluster randomized controlled trial in Zambia
Source: Trials. 2019 Aug 15;20:505. doi: 10.1186/s13063-019-3617-8 (PMC6694552; doi:10.1186/s13063-019-3617-8)

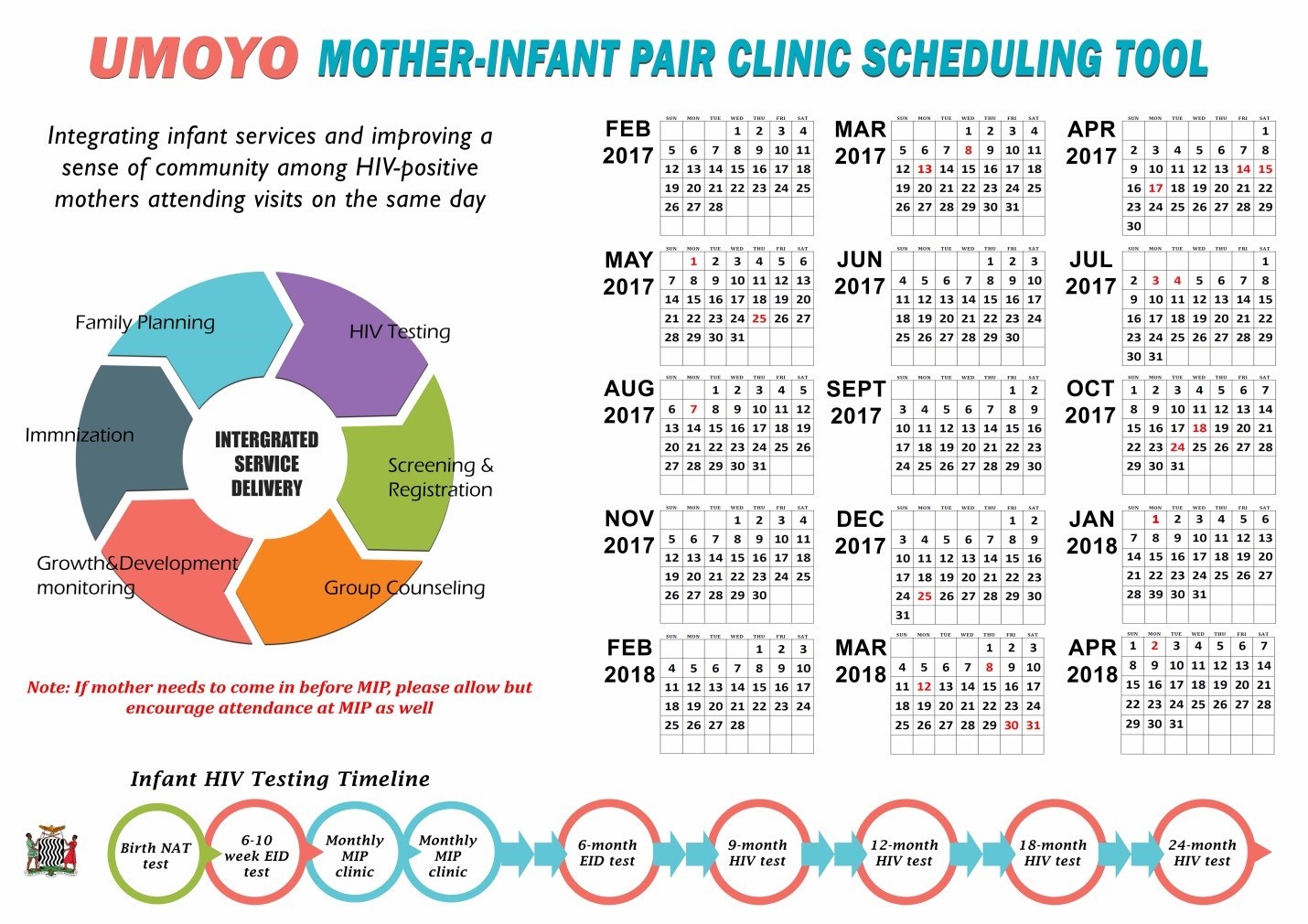

Supplement: Supplementary file 1 — Umoyo scheduling aid. (JPG 355 kb) [file 13063_2019_3617_MOESM1_ESM.jpg]

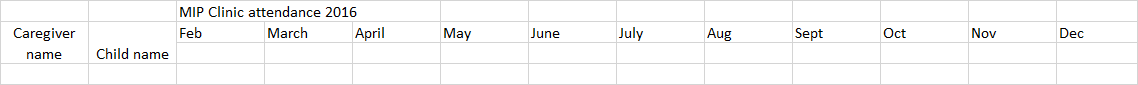

Supplement: Supplementary file 2 — Sample of Umoyo attendance book. (PNG 4 kb) [file 13063_2019_3617_MOESM2_ESM.png]
